# An Empirical Assessment and Comparison of Species-Based and Habitat-Based Surrogates: A Case Study of Forest Vertebrates and Large Old Trees

**DOI:** 10.1371/journal.pone.0089807

**Published:** 2014-02-24

**Authors:** David B. Lindenmayer, Philip S. Barton, Peter W. Lane, Martin J. Westgate, Lachlan McBurney, David Blair, Philip Gibbons, Gene E. Likens

**Affiliations:** 1 Fenner School of Environment and Society, ARC Centre of Excellence for Environmental Decisions and National Environmental Research Program, The Australian National University, Canberra, Australian Capital Territory, Australia; 2 Cary Institute of Ecosystem Studies, Millbrook, New York, United States of America; 3 Department of Ecology and Evolutionary Biology, University of Connecticut, Storrs, Connecticut, United States of America; Bangor University, United Kingdom

## Abstract

A holy grail of conservation is to find simple but reliable measures of environmental change to guide management. For example, particular species or particular habitat attributes are often used as proxies for the abundance or diversity of a subset of other taxa. However, the efficacy of such kinds of species-based surrogates and habitat-based surrogates is rarely assessed, nor are different kinds of surrogates compared in terms of their relative effectiveness. We use 30-year datasets on arboreal marsupials and vegetation structure to quantify the effectiveness of: **(1)** the abundance of a particular species of arboreal marsupial as a species-based surrogate for other arboreal marsupial taxa, **(2)** hollow-bearing tree abundance as a habitat-based surrogate for arboreal marsupial abundance, and **(3)** a combination of species- and habitat-based surrogates. We also quantify the robustness of species-based and habitat-based surrogates over time. We then use the same approach to model overall species richness of arboreal marsupials. We show that a species-based surrogate can appear to be a valid surrogate until a habitat-based surrogate is co-examined, after which the effectiveness of the former is lost. The addition of a species-based surrogate to a habitat-based surrogate made little difference in explaining arboreal marsupial abundance, but altered the co-occurrence relationship between species. Hence, there was limited value in simultaneously using a combination of kinds of surrogates. The habitat-based surrogate also generally performed significantly better and was easier and less costly to gather than the species-based surrogate. We found that over 30 years of study, the relationships which underpinned the habitat-based surrogate generally remained positive but variable over time. Our work highlights why it is important to compare the effectiveness of different broad classes of surrogates and identify situations when either species- or habitat-based surrogates are likely to be superior.

## Introduction

It is financially and logistically impossible to work on the management and conservation of every species in every environment. The complexity of ecosystems has led to the development and use of surrogates to simplify, represent, and help manage complex systems. That is, rather than measuring ecosystems directly, the focus has been on using surrogates as proxies for different ecosystem components such as environmental conditions, habitats, ecological processes, the abundance of particular species, the diversity of particular groups of organisms, or overall biodiversity [1]–[5].

For the purposes of this paper, we use the following overarching definition of a surrogate (modified from [1] and [4]):

“…[a measure] that readily reflects: the biotic or abiotic state of an environment; represents the impact of an environmental change on a habitat, community or ecosystem; the abundance of a particular species; or is indicative of the diversity of a subset of taxa, or of wholesale diversity, within an area.”

Two broad kinds of surrogates used in conservation management are species-based surrogates and habitat-based surrogates. Species-based surrogates are individual species or sets of species, which are used as proxies of diversity within a taxon, or of wholesale diversity within an area or over time. Habitat-based surrogates are habitat attributes that are used as proxies for the presence, abundance or diversity of particular elements or groups of the biota. Habitat-based surrogates are typically based on the structure and/or composition of vegetation at the site level [6]–[8] as well as landscape-level proxies such as the amount and configuration of vegetation cover [9]–[11] or environmental conditions like temperature regimes.

There is a vast and rapidly increasing literature on surrogates (e.g. [1], [3]–[5], [12]–[15]). This massive literature highlights the large demand for, and increasing reliance upon, surrogates in all areas of applied ecology and conservation management. Despite the widespread use of surrogates, their identification and application remains contentious [3], [6], [12], [16]–[21].

A key reason for ongoing debate on surrogates is a paucity of rigorous testing. In particular, few proposed surrogates have been validated [22]–[25] such as by demonstrating that a species-based surrogate accurately predicts the fate of an entity for which it is presumed to be a proxy [13]. Further, there has been limited assessment of the relative effectiveness of different classes of surrogates [1], [7]. Several authors have suggested that habitat-based surrogates are likely to be the most efficient and tractable kinds of surrogates with the greatest practical value for resource managers and policy makers [6], [8]. However, the superiority of habitat-based surrogates has yet to be examined in detail. These problems suggest that considerable work remains to be done to establish the empirical basis for the application of surrogates in ecology.

Rigorous assessment and validation of proposed surrogates is important for several reasons. First, it is vital to ensure that surrogacy relationships have sufficient power to detect key trends in populations or to reflect true levels of environmental change [18], [26]–[29] and, conversely, limit the potential for poor decisions associated with the use of weak or ineffective surrogates. Second, testing is needed to assess the validity of many claims about the effectiveness of surrogates that have typically been made on the basis of assertion, advocacy, and the public appeal of charismatic groups of organisms (reviewed by [30]). Such assertions to date have typically lacked empirical support [3], [4], [31], [32].

In this paper, we present the results of a test of the relative effectiveness of species-based and habitat-based surrogates, using a case study of Australian arboreal marsupials where surrogate use has been contentious. Some researchers have suggested that particular species of arboreal marsupial are **species-based** surrogates [33], in this case meaning that the occurrence of one species will reflect the occurrence of another species, and conserving that (surrogate) species will conserve the broader arboreal marsupial assemblage. An alternative **habitat-based surrogate** for arboreal marsupials is large hollow-bearing trees. Almost all members of the arboreal marsupial assemblage in the wet forests of south-eastern Australia are dependent on large old trees for nesting and denning [34], [35], with some species spending up to 75% of their lives inside such trees. These species do not persist in areas where hollow-bearing trees are absent [34]. Therefore, the abundance of these large old trees could be an appropriate **habitat surrogate** for the presence of arboreal marsupials. While both are legitimate hypotheses, the relative or cumulative usefulness of these two classes of surrogate has not been previously tested.

Our evaluation of both species-based surrogates and habitat-based surrogates was based on a 30-year dataset on arboreal marsupials in the forests of south-eastern Australia. Specifically, we sought to statistically quantify: **(1)** the effectiveness of the abundance of a particular species of arboreal marsupial as a species-based surrogate for other species of arboreal marsupials, **(2)** the effectiveness of the abundance of hollow-bearing trees as a habitat surrogate for the abundance of particular species of arboreal marsupials, **(3)** if a particular class of surrogate was consistently better than the other broad class of surrogate over the 30- year time frame of our work, and **(4)** whether a combination of both species and habitat surrogates performed better than either kind of proxy in isolation.

As part of examining the robustness of species-based and habitat-based surrogates, we modeled not only individual species of arboreal marsupials, but also the overall species richness of animals in this group. We took this approach because estimates of abundance can be an important part of determining the viability of a population of a particular species – for example, animals of conservation concern like the endangered Leadbeater’s Possum (*Gymnobelideus leadbeateri*). Measuring diversity can be important to assess the relative intactness of an assemblage and/or determine whether the occurrence of a species-based surrogate reflects the intactness of the rest of the assemblage of which it is a member. We also wanted to establish whether a particular surrogate was consistently better than the other over the 30- year time frame of our work.

Surrogates will continue to be applied in all ecosystems worldwide [1] and their application will underpin many programs for environmental and biodiversity management and ecological monitoring. The assessment and validation of surrogates is important to avoid problems like inappropriate or weak surrogates [29] which may lead to failed monitoring programs [36], an inability to determine the effectiveness of management interventions [37] or management mistakes [38]–[40]. Our case study provides new insights into the assessment and comparison of different kinds of surrogates and the circumstances in which different classes of surrogates are likely to be superior.

## Methods

### Background: Arboreal marsupials and hollow-bearing trees in the wet forests of Victoria

Our assessment of two kinds of surrogates entailed a case study of arboreal marsupials and large, old, hollow-bearing trees in the montane ash forests of the Central Highlands of Victoria, south-eastern Australia. These forests lie approximately 120 km north-east of Melbourne and cover approximately 60 km×80 km (37°20'–37 ° 55'S and 145° 30'–146° 20'E). Further information on the study area is available in [41].

Montane ash forests have been the focus of ecological studies for three decades [41], [42]. An objective of this research has been to identify ways of conserving arboreal marsupials. There have been long-held concerns about the conservation of marsupials because of the impacts of clearcut logging [41]. Such logging operations have a wide range of effects, including removing large old hollow-bearing trees for periods of 150+ years, thereby significantly impairing the development of suitable habitat for cavity-dependent arboreal marsupials [43]. Furthermore, clearfell logging leads to significant changes in patterns of landscape heterogeneity and fire regimes which also have substantial negative impacts on populations of arboreal marsupials [44], [45].

The arboreal marsupial guild inhabiting montane ash forests comprises eight species: the globally endangered Leadbeater’s Possum, the vulnerable Yellow-bellied Glider (*Petaurus australis*), the Mountain Brushtail Possum (*Trichosurus cunninghami*), Greater Glider (*Petauroides volans*), Sugar Glider (*Petaurus breviceps*), Feathertail Glider (*Acrobates pygmaeus*), Common Ringtail Possum (*Pseudocheirus peregrinus*), and Eastern Pygmy Possum (*Cercartetus nanus*).

No specific permits were required for our field studies as they were observational investigations and no plants or animals were harmed in any way. Permissions to enter the government land where studies were undertaken were issued by Parks Victoria, Melbourne Water, and the Victorian Department of Environment and Primary Industries. All native animal species and native woodland vegetation are protected in Australia, including endangered birds and plants.

### Field data collection

We gathered datasets on the abundance of arboreal marsupials on 1-ha field sites and the abundance of large, old hollow-bearing trees on the same sites. Sites of 1 ha were used in this study because: **(1)** they are broadly congruent with the known home range sizes of our target species [46], and **(2)** such an area is a tractable size for ensuring that all large hollow-bearing trees on a site can be surveyed simultaneously by the stagwatching technique (see below) to give an accurate count of the numbers of individuals of each species (see [42]).

### Data collection for the species-based surrogate

Our species-based surrogate was the abundance of Leadbeater’s Possum on 1-ha field sites, which was a potential proxy for the presence and abundance of other marsupial species, or the species richness of the broader arboreal marsupial guild on a site.

We selected Leadbeater’s Possum as the species-based surrogate for a suite of reasons. In particular, the animal is the target of explicit management action and, for example, has significant areas of forest zones specifically for its conservation [47]. Therefore, management decisions for the species (and by default the rest of the montane ash forest ecosystem) are based on conservation actions undertaken for Leadbeater’s Possum. More generally, Leadbeater’s Possum meets many of the selection criteria as a “landscape species for conservation” (after [48]), such as heterogeneity in its requirements beyond simply a need for large areas, vulnerability, and socio-economic significance (see [41]).

The field method for data collection of the species-based surrogate was “stagwatching” which entails counting the abundance of species of arboreal marsupials on 1-ha sites during a 1-hour period before and after dusk as these animals emerge from large hollow-bearing trees [41]. Stagwatching surveys have been conducted annually since 1983 (except in 1985, 1986, 1995 and 1996) [42] and this field technique proved to be the most accurate method for detecting Leadbeater’s Possum and other species of arboreal marsupials in montane ash forests [41], [49]. We define a hollow-bearing tree in the following section, but note that all hollow-bearing trees on a given site are surveyed simultaneously. Simultaneous stagwatching is necessary for several reasons. First, all species of arboreal marsupials swap regularly between dens in different hollow-bearing trees and failure to stagwatch all hollow-bearing trees at the same time could lead to the same animal being counted multiple times and hence lead to inflated estimates of abundance. In addition, a single hollow-bearing tree may be occupied by any of the species of arboreal marsupials that inhabit montane ash forests, although previous work clearly indicates that different species exhibit a preference for trees with different physical characteristics [50].

The behaviour of arboreal marsupials can be influenced by factors like weather conditions; animals may remain within hollow-bearing trees and not emerge during periods of heavy rain. We controlled for this throughout the 30-year duration of our work by conducting stagwatching surveys only during suitable weather conditions in spring, summer and autumn during times of clear skies, no rain or fog and limited wind.

For the study we report here, we used four datasets collected between 1983 and 2012 in which all field data were gathered in the same way. These were (in time order): **(1)** 146 sites stagwatched between 1983 and 1989 [34], **(2)** 55 sites stagwatched between 1990 and 1993 [35], **(3)** 160 sites stagwatched between 1997 and 2008 and prior to major wildfires in 2009 [42], and **(4)** 63 sites stagwatched between 2009 and 2012 (Lindenmayer et al., unpublished data). None of these 63 sites had been burned in major fires that occurred in 2009. Altogether, there were 218 sites, comprising distinct sets for datasets 1 and 2 plus a further 17 sites which were in dataset 3; dataset 4 was a subset of dataset 3. Dataset 3 had 109 sites in common with dataset 1 and 34 with dataset 2, while dataset 4 had 39 in common with dataset 1 and 19 with dataset 2. During each of these surveys, we gathered data on the abundance of all eight species of arboreal marsupials known to occur in montane ash forests.

### Data collection for the habitat-based surrogate

Our habitat-based surrogate was the abundance of hollow-bearing trees on the same 1-ha sites that were surveyed for Leadbeater’s Possum and other species of arboreal marsupials. A hollow-bearing tree was defined as any stem > 0.5 m in diameter at breast height containing an obvious cavity (as determined by visual inspection with binoculars). We acknowledge that some hollow-bearing trees may not have contained suitable cavities for a particular species of arboreal marsupial at a particular point in time. However, accurate determination as to whether any given tree contained hollows suitable for a given animal is very difficult without completing either a thorough aerial survey of each tree (e.g. by climbing trees to physically inspect hollows) or by felling trees and conducting dissections (although this would then preclude subsequent surveys as the trees are removed from the hollow-bearing tree population).

Each hollow-bearing tree on each site was mapped and geo-referenced with a GPS, and marked using permanent painted numbers and metal tags. This enabled us to readily revisit and re-measure the same hollow-bearing trees on each site over time. Surveys to quantify the collapse and recruitment of hollow-bearing trees, (as well as to model the population dynamics of these trees) were conducted on all sites when they were first established, and were repeated at 3–7 year intervals thereafter (see [43], [51], [52]). Each time a field site was re-surveyed, we completed an additional reconnaissance in which all overstorey eucalypt trees on each site were inspected with binoculars. We completed these surveys to determine if any new cavity trees had been recruited since the previous survey [43]. No additional hollow-bearing trees were recruited to any of our field sites throughout the 30-year duration of our work.

### Statistical analysis

We investigated the four datasets to determine how well a habitat-based surrogate (the number of hollow-bearing trees) and a species–based surrogate (the abundance of Leadbeater’s Possum) predicted the abundance of: **(i)** two target species, the Greater Glider (GG) and the Mountain Brushtail Possum (MBP), as well as how well they predicted **(ii)** the species richness of all arboreal marsupials.

Abundance was measured by counting individual animals on each 1-ha site, and values ranged in our four datasets from 0 to 11 with raw means from 0.3 to 1.1 animals per site. Species richness was measured as the number of species observed at each site, excluding the potential species-based surrogate. Excluding the potential species-based surrogate was appropriate because including it in the count would induce a relationship between the response and explanatory variables in the model simply due to the overlap rather than to any relationship between species. The count ranged from 0 to 4 with raw means from 0.9 to 1.5 species per site.

We modeled the variation of the counts using the negative binomial distribution, as this allowed for over-dispersion due to aggregation effects across sites [53], compared to the simpler Poisson distribution in which variation is a fixed function of the mean. The aggregation parameter is also called the heterogeneity or dispersion parameter, with an infinite value corresponding to the Poisson distribution (see [53]); the variance, *V*, is related to the mean, *m*, by the equation *V*  =  *m* + *m* ^2^/*k*, where *k* is the aggregation parameter.

We used the generalized linear model facilities of the GenStat system to fit the models, and reported the deviance associated with explanatory terms: this is the generalization of sums of squared deviations that is appropriate for the negative binomial distribution [53]. We checked for any evidence of zero-inflation, using the model of [54], but found no significant effect in any of the models fitted for the two chosen target species (but see Section 3.2).

For each of our four survey datasets, and for both of two target species (i.e. GG and MBP), we fitted the explanatory effect of a potential species-based surrogate (the abundance of Leadbeater’s Possum [LP]), a potential habitat-based surrogate (number of hollow-bearing trees [HBT]), and both together. For example, the model with both explanatory variables was as follows, with parameters *a*, *b* and *c*:

log_e_ (Mean abundance of target species)  = 

a + b log_e_ (abundance of LP + 0.5) + c log_e_ (abundance of HBT)

We investigated linearity of each effect using cubic-spline smoothing, extending the models to generalized additive models [55], and compared the actual counts and the log-transformed counts as the explanatory variable. We compared the effect sizes and significance in the three models to evaluate which explanatory variable was more reliably associated with the abundance of the target species. We used the same approach to model species richness.

## Results

### Target species analysis

We found that models using log-transformed counts of either Leadbeater’s Possum or hollow-bearing trees as explanatory variables were nearly always better than those using untransformed counts, in the sense of accounting for more deviance (Table 1, data not shown for tests on the natural scale). Moreover, there was no significant evidence of further non-linearity of the effects of the explanatory variables, except in two cases (discussed below), whereas there were five such cases when using the untransformed counts. We therefore report further results only for the log-transformed counts as explanatory variables. We checked the correlation between the two alternative surrogate variables (transformed), and found it to be moderate: 0.29 (P = 0.004), 0.42 (P = 0.014), 0.10 (P = 0.208) and 0.30 (P = 0.017) for the four datasets, respectively. Therefore, all values were significantly greater than zero (P < 0.02) for all datasets, except for dataset 3.

**Table 1:** Comparison of models using log-transformed counts of the explanatory variables.

			Linear effect				Non-linear effect			
			LP		HBT		LP		HBT	
Dataset	Species	Scale	Deviance	P-value	Deviance	P-value	Deviance	P-value	Deviance	P-value
1	GG	Log	2.95	0.086	14.66	<0.001	0.55	0.457	0.59	0.443
	MBP	Log	9.22	0.002	33.70	0.001	0.12	0.73	0.89	0.35
2	GG	Log	1.01	0.316	37.70	<0.001	0.09	0.770	0.32	0.574
	MBP	Log	0.75	0.386	46.02	<0.001	0.27	0.605	0.61	0.437
3	GG	Log	3.20	0.074	6.51	0.011	0.35	0.555	5.83	0.016
	MBP	Log	2.80	0.094	9.37	0.002	0.52	0.470	2.15	0.143
4	GG	Log	1.18	0.278	16.37	<0.001	0.23	0.632	1.68	0.196
	MBP	Log	0.29	0.593	4.66	0.031	6.69	0.010	0.37	0.542

Comparison of models using log-transformed counts of the explanatory variables (LP abundance of Leadbeater’s Possum, HBT abundance of hollow-bearing trees) fitted individually in negative binomial models for both target species in all four datasets (GG Greater Glider; MBP Mountain Brushtail Possum); the deviance is the change in –2 log(likelihood); the non-linear effect is the difference between a cubic smoothing spline with 2 d.f. and a linear model on the log scale; p-values are approximate because they are based on asymptotic properties.

We found a significant positive association of the abundance of both target species with the habitat-based surrogate in all four datasets, irrespective of whether the species-based surrogate was (or was not) included in the model (Tables 2 and 3). However, the association with the species-based surrogate was significant in only one case (Mountain Brushtail Possum in Dataset 1, in the absence of the effect of the habitat-based surrogate). The values of the regression coefficients, shown in Table 2, are estimated from the model with both log-transformed explanatory variables. This result can be expressed as follows for the example of the model for the Greater Glider in Dataset 1 (see Table 2).

**Table 2:** Estimates of the regression coefficient for target species.

		Habitat-based surrogate				Species-based surrogate			
		GG		MBP		GG		MBP	
Model	Dataset	Estimate	S.e.	Estimate	S.e.	Estimate	S.e.	Estimate	S.e.
Joint	1	0.71^a^	0.20	0.73^a^	0.15	–0.02	0.15	0.15	0.11
	2	1.24^a^	0.24	1.07^a^	0.18	–0.14	0.24	–0.17	0.20
	3	0.40^a^	0.15	0.58^a^	0.20	–0.33	0.17	0.26	0.17
	4	1.78^a^	0.55	0.49^a^	0.24	–0.61	0.42	0.01	0.23
Separate	1	0.70^a^	0.19	0.80^a^	0.14	0.18	0.10	0.34^a^	0.11
	2	1.21^a^	0.24	1.03^a^	0.17	0.34	0.34	0.25	0.30
	3	0.39^a^	0.15	0.59^a^	0.19	–0.30	0.18	0.29	0.18
	4	1.66^a^	0.52	0.49^a^	0.23	–0.32	0.31	0.13	0.23

a Significantly different from 0 at the 5% level.

Estimates of the regression coefficient for the effect of the habitat-based and of the species-based surrogates fitted jointly or separately in negative binomial models for both target species (GG Greater Glider; MBP Mountain Brushtail Possum) in all four datasets.

**Table 3:** Expected abundance of Greater Glider and Mountain Brushtail Possum.

	GG		MBP	
	HBT abundance		HBT abundance	
Dataset	5	10	5	10
1	0.45	0.74	0.73	1.28
2	0.39	0.89	0.74	1.52
3	0.81	1.07	0.42	0.63
4	0.21	0.65	0.78	1.09

Expected abundance of Greater Glider (GG) and Mountain Brushtail Possum (MBP) in each dataset, from the model with a log-linear effect of number of hollow-bearing trees (HBT), for two selected values of the explanatory variable.

Expected no. of GGs  =  0.15× (no. of HBTs)^0.71^ × (no. of LPs + ½)^–0.02^

The quantity 0.15 here is an estimated scaling factor representing the general level of abundance of the Greater Glider for this dataset.

**Figure 1:**
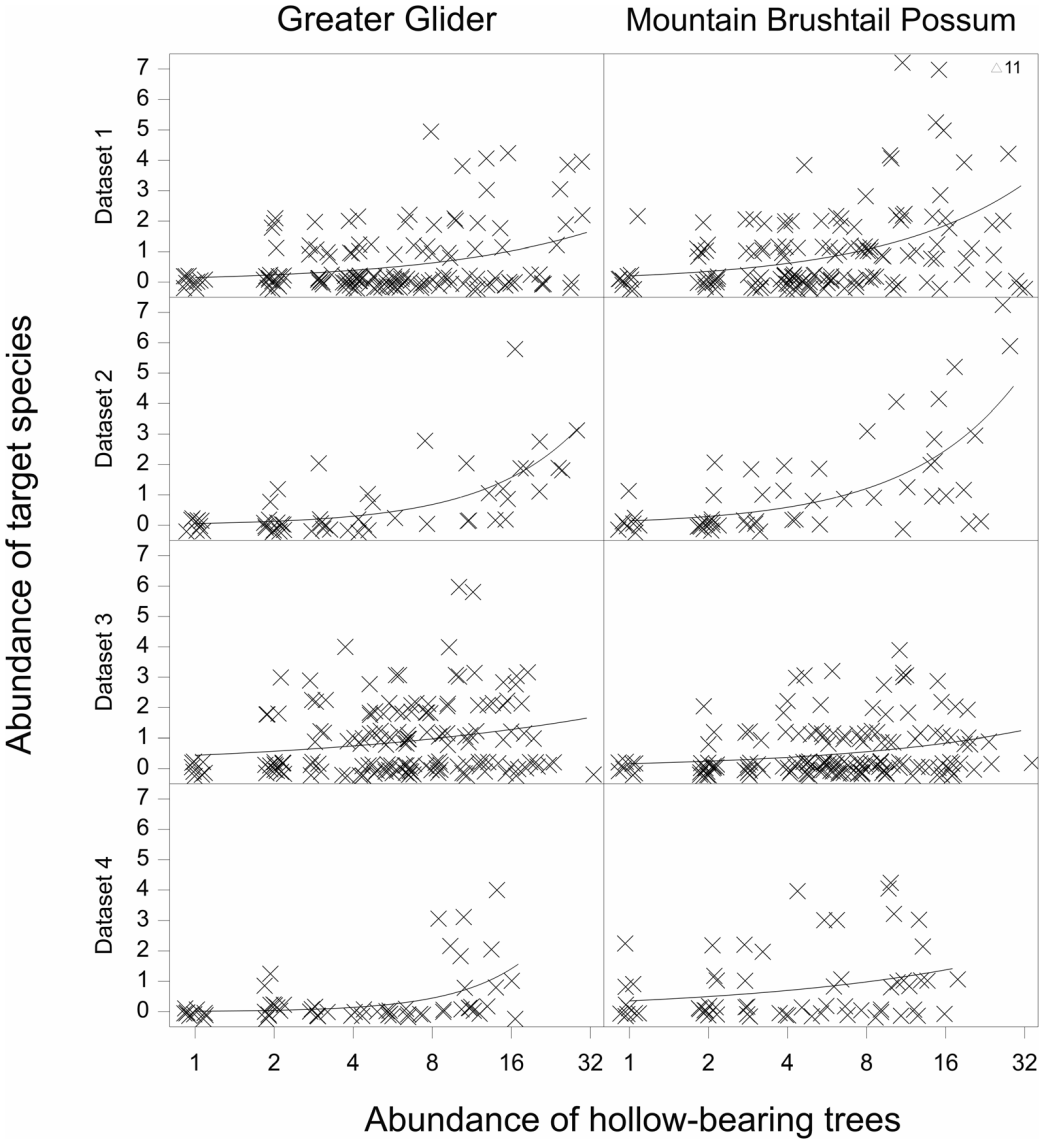
Observed and expected abundance of Greater Glider and Mountain Brushtail Possum. Observed and expected abundance, from the model with transformed counts of hollow-bearing trees as the explanatory variable, for all four datasets. Only one observation was greater than 7 animals, and this is indicated as an abundance of 11 Mountain Brushtail Possums in Dataset 1.

We found that most of the effect of the species-based surrogate was confounded with that of the habitat-based surrogate, while the reverse was not true (Table 2). We therefore summarized the fitted models using just the habitat-based surrogate as the explanatory variable. We illustrate the size of the effect of the abundance of hollow-bearing trees on arboreal marsupial abundance in Table 2 and Figure 1.

As mentioned above, we found evidence of nonlinearity of the effect of the habitat-based surrogate in two cases, even when using log-transformed counts. The first of these was for the abundance of the Greater Glider in Dataset 3, in the model with log abundance of hollow-bearing trees as explanatory variable. This result was attributable to the fact that none of the four sites with the highest number of hollow-bearing trees (20 or more) supported the Greater Glider, which resulted in the smoothed relationship leveling off or even decreasing at the high end of the range of values for the abundance of hollow-bearing trees. The second case was for the abundance of Mountain Brushtail Possum in Dataset 4, in the model with log abundance of Leadbeater's Possum, where only one or no Mountain Brushtail Possums were observed at the eight sites with the highest numbers of Leadbeater's Possum (three or more).

For use in comparison with other analyses using the negative binomial distribution, and for planning new studies of counts of animals like these, we show the estimated values of the aggregation parameter in the fitted models in Table S1. They are mostly near 1.0, except for Dataset 2 where they were over 5.0 with large standard errors. These values make it clear that there was significant aggregation in each dataset, which can be attributed to variation in the underlying mean abundance of the target species among individual sites within a dataset.

### Relationships between two potential surrogates – Leadbeater’s Possum and the abundance of hollow-bearing trees

We fitted models of the abundance of Leadbeater’s Possum using the habitat-based surrogate as an explanatory variable. We found evidence of significant zero-inflation in Datasets 1, 3 and 4, using the zero-inflation model of Lambert [54]. The estimated regression coefficient in the part of this model excluding the extra zeroes, for the transformed explanatory variable (c.f. Table 2, Separate model), was 0.29 (s.e. 0.09), 0.86 (0.33), 0.16 (0.22) and 0.28 (0.27) for the four datasets, of which only the first two were significantly different from zero.

### Robustness of the habitat-based surrogate over time

**Figure 2:**
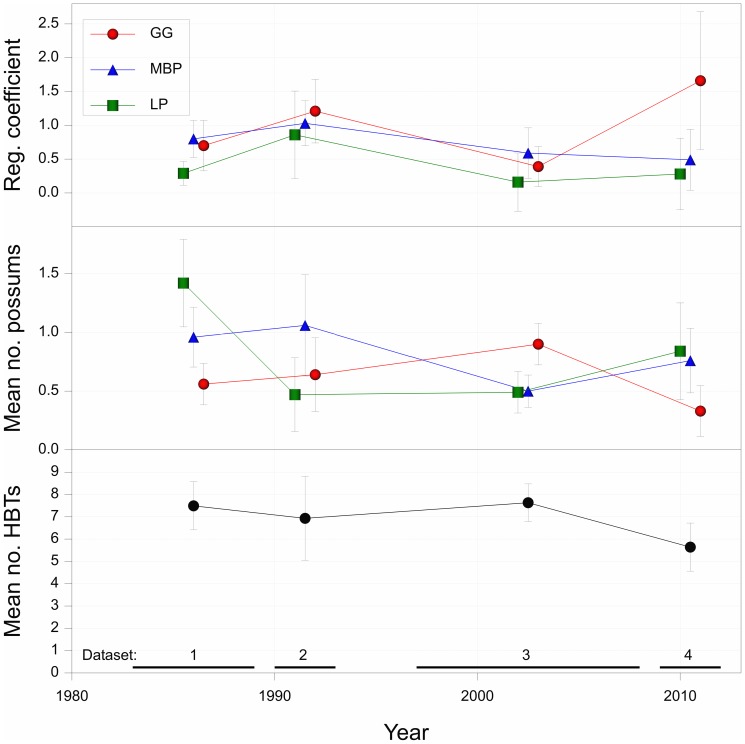
Estimated regression coefficients. Estimated regression coefficients (with standard errors) in the fitted relationship between abundance of three species of marsupial and abundance of hollow-bearing trees, plotted over time, together with the associated mean numbers per site of hollow-bearing trees, and of each species of arboreal marsupial.

We summarized the estimated regression coefficients for all three species of arboreal marsupial from the model using the habitat-based surrogate, plotted against time (the average for each dataset). Our analyses revealed that the association between the habitat-based surrogate and the Greater Glider as well as the Mountain Brushtail Possum was always significant and positive, but the size of the effect differed widely between datasets gathered during different time periods (Figure 2). In the case of Leadbeater’s Possum, the association between the habitat surrogate and the abundance of Leadbeater’s Possum was significant and positive in the first two time periods, but not significantly different from zero in the second two time periods (Figure 2).

### Species richness analysis

We found that once we fitted the effect of the habitat-based surrogate, there was little residual effect of the species-based surrogate in predicting species richness of the whole marsupial assemblage in any of our four datasets (Table 4 and Table S2). This result is similar to our analysis of the Greater Glider and the Mountain Brushtail Possum above. We did find evidence of a positive relationship between species richness and the species-based surrogate in Dataset 1 and Dataset 2, but a non-significant positive relationship in the other two datasets, showing inconsistency in this relationship through time. In all datasets from each time period, the relationship with the habitat-based surrogate was substantially stronger than the species-based surrogate, and there was no evidence of a relationship with the species-based surrogate once the effect of the habitat-based surrogate had been fitted.

**Table 4:** Comparison of negative binomial models for species richness.

	Linear effect				Non-linear effect			
	LP		HBT		LP		HBT	
Data set	Deviance	P-value	Deviance	P-value	Deviance	P-value	Deviance	P-value
1	6.28	0.012	28.68	<0.001	0.68	0.408	1.61	0.204
2	5.62	0.018	32.67	<0.001	0.70	0.404	0.71	0.399
3	0.96	0.327	16.09	<0.001	0.07	0.796	6.05	0.014
4	0.38	0.539	12.83	<0.001	0.98	0.321	0.98	0.322

The comparison uses log-transformed counts of the explanatory variables (LP abundance of Leadbeater’s Possum, HBT abundance of hollow-bearing trees) fitted individually.

## Discussion

### The importance of assessment

Several authors (see [3], [4], [13], [31], [32]) note that the application of surrogates in conservation management has rarely been subjected to detailed assessment (e.g. see [22], [23], [25], [30]). A key part of such assessment is testing of the relative effectiveness of different classes of surrogates [8]. However, to the best of our collective knowledge, no such comparisons have been conducted in terrestrial systems to date (but see [56] for a marine example).

The analyses in the study presented here indicated that the habitat-based surrogate (number of hollow-bearing trees) always had a stronger association with the abundance of the target species (Greater Glider or Mountain Brushtail Possum), and with species richness, than did the species-based surrogate (Leadbeater’s Possum). This outcome was generally consistent with the proposition of other authors who suggested that habitat-based surrogates are likely to perform better than other broad classes of surrogates (e.g. [8]). However, we suggest this proposition needs additional rigorous empirical assessment across a range of different ecosystems.

Based on our empirical assessment, we suggest that the maintenance of populations of the habitat-based surrogate in our case study would be strongly linked to the desired outcome of conserving arboreal marsupials. Indeed, because there has consistently been a very strong statistical association between the habitat-based surrogate and the entities for which it is an intended proxy (see Figure 2; Table 2), we suggest that the probability of a misleading inference arising from the use of this surrogate is low (see [57] for analyses in the context of medical surrogates).

Although the association of the habitat surrogate with the two target species (the Greater Glider and the Mountain Brushtail Possum) was always significant and positive, the size of the effect differed widely between datasets, target species and sites (Table 1; Figure 2). The association between the habitat surrogate and the abundance of Leadbeater’s Possum was significant and positive in the first two time periods, but not significantly different from zero in the second two time periods (Figure 2). This suggests there has been a weakening in the association over time. However, this also could be due to different sets of sites being used in the surveys completed during the four different time points. The reasons for this temporal change remain unclear, although they may be associated with other factors beyond the availability of habitat (e.g. local and landscape-level population dynamics), which can influence animal occurrence. Irrespective of the reasons contributing to the temporal patterns we observed for Leadbeater’s Possum, our findings suggest there will be limitations in using the habitat-based surrogate to predict what would happen over time, or at different locations. We argue that this deficiency should be expected because a measure like the abundance of hollow-bearing trees provides only a partial description of the habitat requirements of a given species [35]; in this case it corresponds only to the nesting resources for arboreal marsupials and not the suitability of, for example, food resources. It is likely that in many cases, even a well-established habitat-based surrogate will, on its own, provide only weak inference. This outcome could also be expected given the inherent complexity that characterizes ecosystems, biotic assemblages and populations, including the suite of factors that comprise the habitat requirements of individual species (see [58]).

### Key issues of efficiency and cost

Important questions that should be addressed as part of the identification of robust surrogates are: is the surrogate logistically efficient, and what are the costs of measuring it? Answering these questions is important (e.g. [59]) as some applications of surrogates appear inefficient: It may require more work to use them appropriately through identifying, rigorously assessing and then correctly applying them, than the direct measurement of the entity for which it is considered indicative [3].

In our case study, surveying nocturnal and mobile arboreal marsupials is time-consuming and labor-intensive. By comparison, it is easier to repeatedly measure the abundance of large hollow-bearing trees. The spatial location and abundance of these trees can sometimes also be identified from the air (e.g. [60]). We estimated measurement effort using experience from the past three decades of field-based empirical work. Based on surveying an average of 45 field sites (each supporting an average of 10 hollow-bearing trees) per year, field surveys of arboreal marsupials would require 450 trees×3 hours per tree  =  1350 hours per year  =  169 person days annually. The cost of a person day is approximately $1000 in 2012 Australian dollars (based on salary and field travel expenses) and this converts to an annual cost of approximately AUD $169 000 for data collection for the species-based surrogate. Field surveys to count the 450 hollow-bearing trees on the same 45 sites would require 15 field days×8 hours per day  =  120 hours per year  =  15 person days per year. This corresponds to an annual cost of $15 000 for the collection of the habitat-based surrogate in 2012 Australian dollars. The habitat-based surrogate was therefore >10 times less effort (and hence substantially less costly) to measure than the species-based surrogate.

### Can ecological processes underlying surrogacy relationships be identified?

The ecological mechanisms driving pattern-based surrogacy relationships are rarely elucidated [1], [3]. In our case study, at least four factors appear to explain habitat-based surrogacy relationships. First, all species of arboreal marsupials in montane ash forests (except the Common Ringtail Possum) nest only in cavities within hollow-bearing trees [61]. Species such as Leadbeater’s Possum spend up to 75% of their lives within a cavity inside a hollow-bearing tree [62]. Second, all species of arboreal marsupials swap regularly between dens and nests in different hollow-bearing trees [63]. Therefore, only sites with numerous hollow-bearing trees will meet these behavioral requirements. Third, different species of arboreal marsupials tend to select hollow-bearing trees with different external characteristics (e.g. size, height, cavity diameter, levels of decay), suggesting inter-specific resource partitioning [46]. Fourth, different species of arboreal marsupials rarely co-occupy the same hollow-bearing tree [64]. Thus, many hollow-bearing trees are required to meet the demands of multiple species of arboreal marsupials within a site.

The interaction between hollow availability and inter-specific competition for hollows is a likely mechanism underpinning the surrogacy relationships we describe. Although we found that the occurrence of one marsupial species was often positively associated with the second species, this association was relatively small and sometimes became negative once the habitat surrogate was included in the model. This result implies that competitive exclusion may be occurring across some sites where hollow resources are limiting. While this is not unexpected from an ecological perspective, it highlights that care must be taken to elucidate the mechanistic basis of any proposed ecological surrogates, and consider the conditions under which those assumptions are likely to hold [1]. Furthermore, we acknowledge that an important caveat associated with this study was that some hollow-bearing trees may not have contained suitable cavities for particular species at a particular point in time. We surveyed all hollow-bearing trees on all sites as a prelude to stagwatching all species of arboreal marsupials on those same sites. However, tree size, tree condition, cavity dimensions and other factors may have resulted in some trees being inappropriate for occupancy by some species [50]. Therefore, the total abundance of all hollow-bearing trees on a site cannot be a complete proxy for the presence and/or abundance of all species of arboreal marsupials nor for the overall species richness of these animals.

### Temporal reliability

The vast majority of studies of surrogates in ecology and conservation have been snapshot investigations [1] with few assessments of temporal robustness [30]. Our analyses indicated that the habitat-based surrogate generally remained positive throughout the 30-year period of the datasets used in assessment, although the size of the effect differed widely between datasets (Figure 2). However, we do not assume the abundance of hollow-bearing trees will **always** be a reliable habitat surrogate in montane ash forests. A particular risk is a change in the temporal strength of the surrogacy relationship; as we observed in the case of the Greater Glider (Figure 2). This weakening might occur as the abundance of hollow-bearing trees declines over time (see [65]), and there is a behavioral shift among animals to such changes in nesting resources. It also may arise because other processes like patch- and landscape-level population dynamics are altered, leaving sites with otherwise apparently suitable habitat unoccupied by arboreal marsupials.

### Spatial and taxonomic boundaries for surrogacy relationships

An important part of the application of surrogates is to define the spatial and taxonomic boundaries within which particular kinds of surrogate relationships are valid, but beyond which their application is invalid and has a high risk of failure and associated management errors (see [19]). The spatial boundaries of the habitat-based surrogate in our case study are clear; they are restricted to the montane ash forests of the Central Highlands of Victoria, Australia. The relationships between the abundance of hollow-bearing trees and the presence and abundance of arboreal marsupials described above have **not** been identified elsewhere in south-eastern Australia [66], [67]. The taxonomic boundaries of our habitat-based surrogate are also clear; they are limited to the arboreal marsupials in montane ash forests. Relationships between the abundance of animals and the abundance of hollow-bearing trees are not found for other groups like birds [68] or small terrestrial and scanscorial mammals in the montane ash forests of Victoria [69].

### Under what circumstances will a given class of surrogate be superior?

A key question in the application of surrogates is: When will one kind of surrogate be better than another? Based on the work we report here, we suggest that if the habitat requirements of a given species were unknown or if there were very close links between two or more taxa (e.g. a symbiotic or tight mutualistic relationship), then a species-based surrogate would be superior. Conversely, we believe that a habitat-based surrogate is likely to be superior when the habitat requirements of a species are well known and there is a readily measured limiting resource that is part of those well-known habitat requirements. This approach would enable measurements of that limiting resource to be used as a habitat-based surrogate. There are many examples worldwide where a key element structural or floristic element of the vegetation can be readily measured and is known to be an important limiting resource for a species or suite of taxa. Examples extend beyond large old trees (e.g. [70], [71]; reviewed by [72]) to include [73], [74], large pieces of coarse woody debris [75] and host plants for herbivorous insects (e.g. [76]). However, if a given resource were not limiting, then measurements of that resource would be unlikely to act as a particularly robust habitat-based surrogate. In addition, we suggest that the effectiveness of a habitat-based surrogate may be curtailed for rare species that may be absent from areas of apparently suitable habitat due to the influence of factors beyond solely the availability of habitat. Finally, some particular habitat attributes may be extremely difficult to measure (unlike the general category of hollow-bearing tree in our study) and in such cases, a species-based surrogate may be more readily and cost-effectively measured and hence superior.

## Concluding Remarks

Surrogates will undoubtedly continue to be a major component of conservation management well into the future (reviewed by [1]). Therefore, more rigorous assessment of surrogates, as well as comparing broad classes of surrogates is badly needed. This assessment can reveal which kinds of surrogates are statistically robust, comparatively superior as well as efficient and less expensive to measure. This work, in turn, helps determine kinds of surrogates that will perform best in particular monitoring programs and in helping to best quantify the effectiveness of conservation management interventions.

## Supporting Information

Table S1 **Estimates of the aggregation parameter for target species.**
(DOC)

Table S2 **Estimates of the regression coefficient for species richness.**
(DOC)
