# Supplementary material for: An Empirical Assessment and Comparison of Species-Based and Habitat-Based Surrogates: A Case Study of Forest Vertebrates and Large Old Trees
Source: PLoS One. 2014 Feb 24;9(2):e89807. doi: 10.1371/journal.pone.0089807 (PMC3933686; doi:10.1371/journal.pone.0089807)
Supplement: Table S1 — Estimates of the aggregation parameter for target species. (DOC) [file pone.0089807.s001.doc]

**Table S1: Estimates of the aggregation parameter for target species**

Estimates of the aggregation parameter in the negative binomial models for the effect of the habitat-based surrogate for both target species (GG Greater Glider, MBP Mountain Brushtail Possum).

|  | GG | | | MBP | |
| --- | --- | --- | --- | --- | --- |
| Dataset | | Estimate | S.e. | Estimate | S.e. |
| 1 | | 0.69 | 0.26 | 1.42 | 0.47 |
| 2 | | 5.92 | 9.93 | 5.30 | 6.25 |
| 3 | | 1.45 | 0.53 | 1.10 | 0.50 |
| 4 | | 0.65 | 0.50 | 0.86 | 0.45 |
