# Supplementary material for: An Empirical Assessment and Comparison of Species-Based and Habitat-Based Surrogates: A Case Study of Forest Vertebrates and Large Old Trees
Source: PLoS One. 2014 Feb 24;9(2):e89807. doi: 10.1371/journal.pone.0089807 (PMC3933686; doi:10.1371/journal.pone.0089807)
Supplement: Table S2 — Estimates of the regression coefficient for species richness. (DOC) [file pone.0089807.s002.doc]

**Table S2: Estimates of the regression coefficient for species richness**

Estimates of the regression coefficient for the effect of the habitat-based and of the species-based surrogates fitted jointly or separately in negative binomial models for species richness.

|  |  | Habitat-based surrogate | | Species-based surrogate | |
| --- | --- | --- | --- | --- | --- |
| Model | Dataset | Estimate | S.e. | Estimate | S.e. |
| Joint | 1 | 0.48^a^ | 0.10 | 0.06 | 0.08 |
|  | 2 | 0.80^a^ | 0.16 | 0.06 | 0.18 |
|  | 3 | 0.44^a^ | 0.12 | 0.07 | 0.11 |
|  | 4 | 0.60^a^ | 0.18 | –0.05 | 0.16 |
| Separate | 1 | 0.51^a^ | 0.10 | 0.19^a^ | 0.07 |
|  | 2 | 0.81^a^ | 0.15 | 0.44^a^ | 0.18 |
|  | 3 | 0.44^a^ | 0.11 | 0.11 | 0.11 |
|  | 4 | 0.58^a^ | 0.17 | 0.10 | 0.15 |

^a^ Significantly different from 0 at the 5% level.
